# A novel recombinant lineage’s contribution to the outbreak of coxsackievirus A6-associated hand, foot and mouth disease in Shanghai, China, 2012-2013

**DOI:** 10.1038/srep11700

**Published:** 2015-06-30

**Authors:** Xiaobo Feng, Wencai Guan, Yifeng Guo, Huiju Yu, Xiaoling Zhang, Ruhong Cheng, Zhen Wang, Zhen Zhang, Jia Zhang, Huaguo Li, Yin Zhuang, Hui Zhang, Zhiyong Lu, Ming Li, Hong Yu, Yixiao Bao, Yunwen Hu, Zhirong Yao

**Affiliations:** 1 Department of Dermatology, Xinhua Hospital Affiliated to Shanghai Jiaotong University School of Medicine, Shanghai, China; 2 Pathogen Diagnosis and Biosafety Department, Shanghai Public Health Clinical Center, Key Lab of Medical Molecular Virology, Fudan University, Shanghai, China; 3 Department of Pediatrics, Xinhua Hospital Affiliated to Shanghai Jiaotong University School of Medicine, Shanghai, China

## Abstract

Since late 2012, coxsackievirus A6 (CVA6) has gradually become the predominant pathogen responsible for hand-foot-mouth disease (HFMD) in several provinces of China. A total of 626 patients diagnosed with HFMD in Shanghai, China from January 2012 to September 2013 were enrolled in this study. Of these, 292 CVA6 infected cases were subjected to clinical analyses. Whole-genome sequencing, recombination and phylogenetic analyses were also performed. A recombinant CVA6 monophyletic lineage was found during an outbreak of CVA6-associated HFMDs in Shanghai, China in November 2012, and accounted for 21.9% (64/292) of the CVA6 strains during the study period. Recombination analyses showed that the 2C gene of the novel CVA6 virus was probably derived from a coxsackievirus A4 (CVA4) strain circulating in the population. Clinical observation showed that this recombinant CVA6 virus led to a more generalized rash than did the non-recombinant CVA6 virus. This newly emerged CVA6 lineage was associated with a considerable proportion of HFMD cases from 2012 to 2013 in Shanghai, and poses a potential threat to public health.

The clinical manifestations of hand-foot-month disease (HFMD) include mild to severe skin rash, pulmonary edema, circulatory disorders, menigoencephalitis, aseptic encephalitis, and even death. Large outbreaks in the Asia-Pacific region have been described since 1997, and it is a common epidemic disease around the world^1^,^2^. During the last few years, large-scale outbreaks of HFMD have occurred in China^3^,^4^,^5^,^6^, and have become a significant public health issue. From January 2012 to September 2013, a total of 3,686,764 cases of HFMD of which 792 were fatal were reported in mainland China according to the Chinese Center for Disease Control and Prevention (http://www.chinacdc.cn/tjsj/fdcrbbg/).

The causative agents of HFMD are human enteroviruses (EVs) belonging to genus *Enterovirus*, family *Picornaviridae*. Enterovirus A (EV-A) species was the main pathogen for HFMD, followed by Enterovirus B (EV-B). EV-A (formerly named Human enterovirus A) consists of 25 serotypes: coxsackievirus A2 (CV-A2), CV-A3, CV-A4, CV-A5, CV-A6, CV-A7, CV-A8, CV-A10, CV-A12, CV-A14, CV-A16, enterovirus A71 (EV-A71), EV-A76, EV-A89, EV-A90, EV-A91, EV-A92, EV-A114, EV-A119, EV-A120, EV-A121 and the simian enteroviruses SV19, SV43, SV46 and baboon enterovirus A13 (BA13) (www.picornaviridae.com). The genome of EV is composed of a single open reading frame encoding a polyprotein which is cleaved by viral proteases into the mature viral capsid proteins P1 (VP4, VP2, VP3, and VP1) and non-structural proteins P2 (2A to 2C) and P3 (3A to 3D)^7^. Enteroviruses generate genetic diversity and evolve through nucleotide mutations or genetic recombination. Epidemiologic studies have indicated that distinct enteroviruses could recombine with each other^5^,^8^,^9^. Inter-typic recombinant EVs were reported to cause severe or fatal cases of HFMD^10^,^11^; however, there was no clear evidence that inter-typic recombinant EVs had previously contribute to outbreaks of HFMDs^5^,^12^.

Historically, EV71 and CVA16 were reported to be the major etiological agents of HFMD worldwide^7^. Since 2008, CVA6 has emerged gradually and caused atypically generalized and sometimes severe exanthema in HFMD cases around the world^13^,^14^. In 2008, an outbreak of HFMD caused by CVA6 was first reported in Finland and similar outbreaks were then reported worldwide^13^,^15^,^16^,^17^,^18^,^19^,^20^,^21^,^22^,^23^,^24^. Since late 2012, CVA6 has gradually become the predominant pathogen responsible for HFMD in several provinces of China^21^,^25^,^26^,^27^.

Because the number of HFMD cases with generalized skin lesions has increased in Shanghai, China since Nov 2012, we performed molecular identification of enteroviruses from HFMD cases, whole-genome sequencing, recombination and phylogenetic analysis, as well as a determination of clinical relevance.

## Methods

### Patients, specimens and data collection

From January 2012 to September 2013, a total of 626 patients were diagnosed with HFMD by the Xinhua Hospital affiliated to Shanghai Jiaotong University School of Medicine according to diagnostic criteria defined by the National Health and Family Planning Commission of the People’s Republic of China (http://www.nhfpc.gov.cn/yzygj /s3593g/201306/6d935c0f43cd4a1fb46f8f71acf8e245.shtml), and 1251 clinical specimens (626 throat swabs, 71 stools, 28 vesicle swabs, 526 sera) were collected from these patients. The distribution of the patients covered all 18 municipal districts and one county in Shanghai. This study was approved by the Ethics Committee of Xinhua Hospital, and the procedures were carried out in accordance with approved guidelines. Informed consent was obtained from the subjects’ parents or guardians.

Patients who met the following 3 criteria were selected for clinical correlation analysis: 1) under 14 years of age, 2) skin lesions compatible with typical HFMD or atypical HFMD, 3) CVA6 identified by virological methods. The skin lesions of typical HFMD are commonly manifest as small vesicles, papulovesicular lesions or macular rashes on the palms, soles, buttocks and oral mucosa. Atypical HFMD is manifest as large vesicles or bullae, maculopapular rashes or target-like lesions presenting on any site of the body including the trunk, limbs or facial areas, as well as symptoms of acute viral infection such as fever, cough or diarrhea. Patients who met any of the following conditions were excluded in the analyses: 1) lesions localized on the oral mucosa, particularly the soft palate or tonsillar pillars, without involvement of any other sites in the body, which were diagnosed as herpangina; 2) immune-compromised subjects with a history of systemic illness who received chemotherapy or immunosuppressive treatment; 3) non CVA6 viruses detected by virological analysis.

Demographic data, clinical manifestations, and laboratory findings were recorded. Among the clinical manifestations, fever, timing of skin lesions, distribution of skin lesions including the generalized form, and systemic symptoms were evaluated. According to the number of anatomical sites involved (9 sites: hands, feet, buttocks, oral mucosa, upper limbs, lower limbs, anterior trunk, posterior trunk and face/neck), the generalized form of HFMD was defined as skin lesions at five or more of these sites^28^. Laboratory findings including complete blood count, biochemistry profile, cardiac enzymes, chest X-ray, electrocardiogram (EKG), electroencephalogram (EEG) and MRI of the brain were performed.

### Serotypic identification of enteroviruses

Clinical specimens including throat swabs, vesicular fluids, feces and sera were collected from HFMD patients for laboratory examinations. Viral RNA was extracted directly from clinical specimens using a QIAamp Viral RNA Mini Kit (Qiagen, Santa Clara, CA) and stored at −80 °C. The identification of EV and serotyping of EV71 and CVA16 from samples were performed by a real-time reverse transcription polymerase chain reaction (RT-PCR) as previously described^29^,^30^. To further identify the EV serotypes other than EV71 and CVA16, semi-nested RT-PCR and sequencing were conducted as previously described^31^. Serotype was determined by comparison of the viral sequences with corresponding sequences of the EV prototype strains using blastn online (http://blast.ncbi.nlm.nih.gov/ Blast.cgi).

### Complete genome amplification and sequencing

Complete genome amplification and sequencing were performed using viral RNA extracted directly from clinical specimens collected during pre-outbreak and outbreak of CVA6-associated HFMDs. Fourteen CVA6s, including pre-outbreak strains of 1827/CVA6/SH/CHN/2011, 1232/CVA6/SH/CHN/2010, 3913/CVA6/SH/CHN/2011 and 4368/CVA6/SH/CHN/2012, outbreak strains of 5039/CVA6/SH/CHN/2013, PF3/CVA6/SH/CHN/2013, 5056/CVA6/SH/CHN/2013, 4592/CVA6/SH/CHN/2012, 5068/CVA6/SH/CHN/2013, 4645/CVA6/SH/CHN/2012, PF19/CVA6/SH/CHN/2013, 5047/CVA6/SH/CHN/2013, PF001/CVA6/SH/CHN/2013 and 5084/CVA6/SH/CHN/2013, together with two recent strains of CVA4 (701/CVA4/SH/CHN/2010 and 1047/CVA4/SH/CHN/2010) circulating in the Shanghai area were randomly selected and subjected to complete genome amplification using primers listed in Supplemental Table 1. Overlapping fragments covering the viral genome were amplified using a one-step RT-PCR kit (TaKaRa, Dalian, China), and primers were designed based mainly on the recently emerging CVA6 and CVA4 genome sequences published in previous studies^32^,^33^. The non-structural proteins in the P2 and P3 regions of partial CVA6 strains were determined by primer walking strategy in which additional primers were designed on the basis of the initial PCR products for further amplification. One-step RT-PCR was performed with a final volume of 50 μL containing 5 μL genomic RNA, 25 μL 2×one step RT-PCR buffer, 1 μL TaKaRa Ex Taq HS, 1 μL PrimeScript RT Enzyme Mix, 1 μL of each primer (10 μM), and 16 μL RNase Free dH2O. RT-PCR was performed in an Eppendorf Mastercycler at 42 °C for 15 min, 95 °C for 1 min, followed by 40 cycles of 95 °C for 20 s, 56 °C for 30 s, and 72 °C for 1.2 min with a final extension step at 72 °C for 10 min. The PCR products were purified and sequenced in both directions on an automated sequencer ABI 3730XL (Applied Biosystems, Foster City, CA). The sequenced DNA fragments were assembled into complete genomes using ContigExpress project in Vector NTI version 11.5. Sequence similarities among strains were calculated using BioEdit 7.2.5. The full length genomic sequences indicated above were deposited in GenBank under the accession numbers KJ541154-KJ541169. All 14 CVA6 genome sequences were used in phylogenetic analysis based on VP1, 2C and 3D region.

### Recombination analysis

Thirteen full-length genome sequences of recently emerging EV-A strains including 5039/CVA6/SH/CHN/2013, PF3/CVA6/SH/CHN/2013, 5056/CVA6/SH/CHN/2013, 4592/CVA6/SH/CHN/2012, 5068/CVA6/SH/CHN/2013, 4645/CVA6/SH/CHN/2012, PF19/CVA6/SH/CHN/2013, 5047/CVA6/SH/CHN/2013, 5084/CVA6/SH/CHN/2013, 701/CVA4/SH/CHN/2010 and 1047/CVA4/SH/CHN/2010 detected in the present study, together with HQ728260/CVA4/GD/CHN/2009 and JF799986/EV71/GD/CHN/2009 (a progeny derived from the recombination of EV71 subgenotype C4 and CVA4, which was closely related to recombinant CVA6 in the non-structural protein region)^34^ which are available in GenBank were aligned with 12 prototypes including CVA2~8,10,12,14,16,EV71 which were considered to be the most closely related strains of EV-A based on non-structural protein region (Supplemental Table 2). The sequence alignment was performed using ClustalW2 as depicted previously^5^, and then similarity plots among the aligned sequences were generated using SimPlot version 3.5.1. Similarity was calculated in each window of 200 bp by the Kimura (2-parameter) distance model with a transition-transversion ratio of 2.0. The window was successively advanced along the genome alignment in 20 bp increments. For bootscan analysis, the neighbor-joining algorithm was run with 500 bootstrap replicates. A threshold of 70% or more of the observed permuted trees indicated potential recombination events. A genetic algorithm for the detection of a recombination (GARD) program (http://www. datamonkey.org/dataupload.php) was then used to confirm recombination and search for putative breakpoints with a phylogenetic incongruence test in which the HKY85 nucleotide substitution bias model was selected. Support for recombination was based on AICc goodness of fit and the KH test for phylogenetic incongruence. Homologous sequences corresponding to diverse parts of the genome from the recombinant CVA6 were searched within GenBank using blastn. The sequence homology of diverse parts of the genome between the recombinant CVA6 and the closely related prototype and recently emerging EV-A strains were calculated separately by BioEdit.

### Differentiation of recombinant and non-recombinant CVA6 strains by type-specific RT-PCR

To determine the proportion of recombinant and non-recombinant CVA6 strains in this surveillance, a one-step duplex RT-PCR assay targeting partial 2B and 2C protein gene with primers specific to recombinant CVA6 and non-recombinant CVA6 strains, respectively (Supplemental Figure 1), was developed and applied for differentiation of all CVA6 isolates involved in this study. The duplex RT-PCR was performed using primers RCA6F, RCA6R, NRCA6F and NRCA6R (Supplemental Table 1) by which recombinant CVA6 yielded 222-bp amplicon and non-recombinant CVA6 yielded 543-bp amplicon. The reaction volume of 50 μL contained 5 μL genomic RNA, 25 μL 2 × one step RT-PCR buffer, 1 μL TaKaRa Ex Taq HS, 1 μL PrimeScript RT Enzyme Mix, 1 μL of each primer (10 μM), and 16 μL RNase Free dH2O. RT-PCR was performed in an Eppendorf Mastercycler at 42 °C for 15 min, 95 °C for 1 min, followed by 40 cycles of 95 °C for 20 s, 56 °C for 30 s, and 72 °C for 30 s with a final extension step at 72 °C for 7 min. PCR products were separated on a 1.5% (wt/vol) agarose gel at 140 V for 25 min. Recombinant CVA6 isolates identified by duplex RT-PCR were further confirmed using sequencing of the 2C partial region. Additionally, a CVA4 type-specific PCR was employed to rule out the mixed infection with CVA4 in samples in which recombinant CVA6 was identified (see Supplemental Table 4).

### Amplification and sequencing of VP1, 2C and 3D genes

The complete VP1 gene was amplified and sequenced using primers 2333CA6F and 3530CA6R (Supplemental Table 1). The one-step RT-PCR was performed in a final volume of 50 μL containing 5 μL genomic RNA, 25 μL 2×one step RT-PCR buffer, 1 μL TaKaRa Ex Taq HS, 1 μL PrimeScript RT Enzyme Mix, 1 μL of each primer (10 μM), and 16 μL RNase Free dH2O. RT-PCR was performed in an Eppendorf Mastercycler at 42 °C for 15 min, 95 °C for 1 min, followed by 40 cycles of 95 °C for 20 s, 56 °C for 30 s, and 72 °C for 1.2 min with a final extension step at 72 °C for 10 min.

The 5′ region of 2C protein gene after the breakpoint was amplified and sequenced with primers 3714CA6F and 4579CA6R (Supplemental Table 1). The one-step RT-PCR mixture is the same as stated above. RT-PCR was performed in an Eppendorf Mastercycler at 42 °C for 15 min, 95 °C for 1 min, followed by 40 cycles of 95 °C for 20 s, 56 °C for 30 s, and 72 °C for 50 s with a final extension step at 72 °C for 10 min.

The partial 3D gene was amplified and sequenced with primers 3DCA6F and 3DCA6R (Supplemental Table 1). The one-step RT-PCR mixture is the same as stated above. RT-PCR was performed in an Eppendorf Mastercycler at 42 °C for 15 min, 95 °C for 1 min, followed by 40 cycles of 95 °C for 20 s, 56 °C for 30 s, and 72 °C for 1 min with a final extension step at 72 °C for 10 min.

The complete VP1 gene was amplified and sequenced for 61 recombinant and 101 non-recombinant CVA6 strains. The 61 recombinant CVA6 and 33 randomly selected non-recombinant CVA6 strains were amplified and sequenced for the partial 2C and 3D genes. The PCR products were purified and sequenced on an automated sequencer ABI 3730XL (Applied Biosystems, Foster City, CA).

The VP1, 2C and 3D sequences obtained in this study were deposited in GenBank under the accession numbers JX495118-JX495149, KC414726-KC414757, KF647875-KF647892, KJ541170-KJ541441, KP398832-KP398846.

### Phylogenetic analysis

The capsid protein VP1 (915 bp), non-structure proteins 2C (425 bp) and 3D (812 bp) fragments were selected for phylogenetic analyses. Dendrograms were produced by neighbor-joining analysis using sequences alignment with the Kimura 2-parameter method. Gaps were treated as a complete deletion. Statistical support for each clade was assessed using bootstrap analysis with 500 replicates. Genetic distances were calculated with the p-distance model.

The 61 recombinant CVA6 and 33 randomly selected non-recombinant CVA6 strains together with EV-A prototypes (CVA2~8, 10,12,14,16, EV71) and closely related EV-A strains were subjected to phylogenetic analyses based on VP1, 2C and 3D regions, respectively. The reference strains of EV used in 3 phylogenetic trees were retrieved from the Genbank database, which shared the highest identity with our sequences by using BLAST tools.

Additionally, the 162 CVA6 strains including 61 recombinant and 101 non-recombinant CVA6 strains in our study and other CVA6 strains worldwide available from GenBank were subjected to phylogenetic analysis based on full length sequence of the VP1 gene.

### Statistical analyses

Discrete variables were expressed as counts (percentage) and continuous variables were expressed as means ± standard deviation (SD). Variations between groups were evaluated using the T-test for age, duration of fever and body temperature. Differences for discrete variables (gender, contact history, timing of skin lesion, clinical characteristics and laboratory data) were analyzed by using the chi-square test or Fisher’s exact test. *P* < 0.05 was considered to be statistically significant.

## Results

### Serotyping of entroviruses associated with HFMD cases

**Figure 1:**
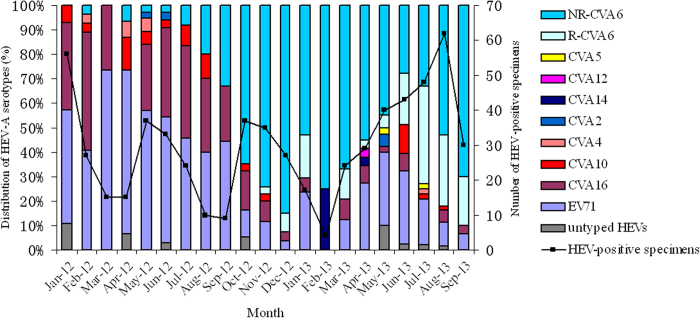
Monthly distribution of EV-A in Shanghai, China, from January 2012 to September 2013. The continuous line describes the total number of specimens collected each month in Shanghai; the histogram depicts the distribution of recombinant CVA6 (R-CVA6), non-recombinant CVA6 (NR-CVA6) and other EV-A serotypes identified each month.

From January 2012 to September 2013, a total of 626 patients with a clinical diagnosis of HFMD were investigated in this study. A definite serotype was identified in 609 (97.3%) cases and the most common EV-A was CVA6, which accounted for 292 (46.6%) cases, followed by EV71 (181; 28.9%), CVA16 (96; 15.3%), CVA10 (22; 3.5%), CVA4 (5; 0.8%), CVA2 (4; 0.6%), CVA5 (2; 0.3%), and CVA12 (1; 0.2%). Before September 2012, EV71 was the dominant serotype in HFMD cases (115; 50.9%), whereas CVA6 dominated (281; 70.3%) after October 2012 (Fig. 1).

### Full length genomic sequencing and recombination analysis of the recently emerging CVA6 isolates

To elucidate the molecular features of prevalent CVA6 strains causing the outbreak of CVA6-associated HFMDs, fourteen CVA6 strains from 14 cases before and during the CVA6 outbreak period were randomly selected and the whole genome of each virus was amplified directly from the clinical specimens. Sequences alignment throughout the whole genome showed that, 5039/CVA6/SH/CHN/2013, PF3/CVA6/SH/ CHN/2013 and 5056/CVA6/SH/CHN/2013, which were collected during the CVA6 outbreak period, exhibited significant sequence divergence from all the previously published non-recombinant CVA6s in the noncapsid protein region. The identities of a representative strain (PF3/CVA6/SH/CHN/2013) and other EV stains are shown in Table 1. PF3/CVA6/SH/ CHN/2013 had the highest sequence identity (94.2% ~ 96.7%) with non-recombinant CVA6 strains in the 5′ UTR, VP4, VP2, VP3, VP1 and 2A regions; however the similarity dropped to 87.8% and 82.9% in the 2B and 3A regions, respectively. Notably, in the 2C, 3B, 3D and 3′ UTR regions, this CVA6 strain was more similar to the other EV-A serotypes than to the non-recombinant CVA6 strains, suggesting that a recombinant event occurred with these viruses (selected isolates which are closely related to recombinant CVA6 are shown in Table 1). The P3 region of the recombinant CVA6 virus showed less than 91% of similarity with other sequences including CVA6 strains available from GenBank.

**Figure 2:**
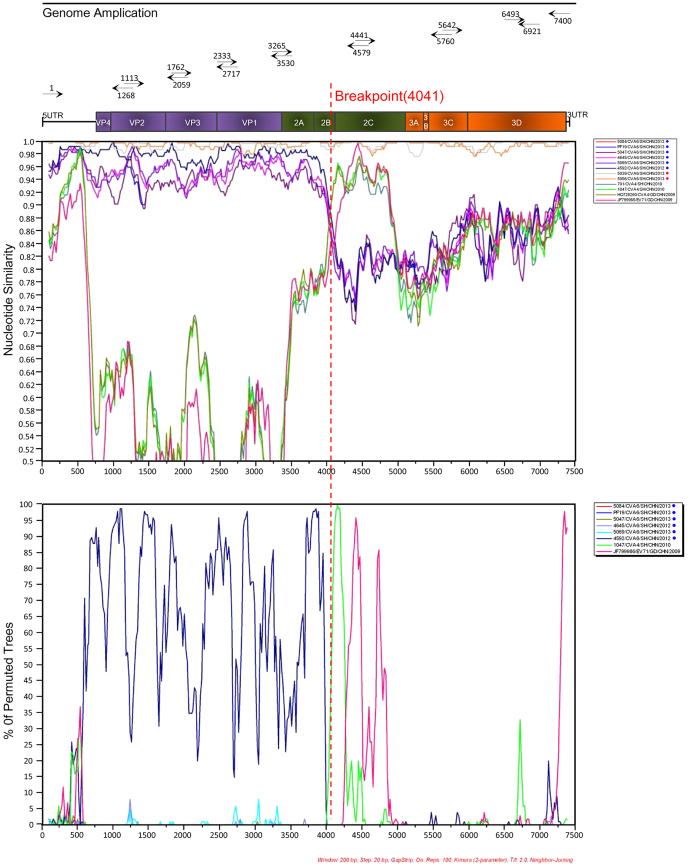
Simplot and Bootscanning analyses of the recombinant CVA6, non-recombinant CVA6, recombinant EV71 and recently emerging CVA4 strains on the basis of full-length genomes. Recombinant CVA6 (PF3/CVA6/SH/CHN/2013) was used as the query sequence. Recombinant CVA6 and non-recombinant CVA6 strains were marked by red dots and blue dots, respectively. This is a schematic representation of the amplified regions of the CVA6 genome. Primer names and binding sites are shown by short arrows.

To corroborate our hypothesis, Simplot analysis and subsequent bootscan analysis were employed (Fig. 2 shows only the most closely related isolates). It was found that recombination has probably occurred at the 2C regions. The putative recombinant fragment showed highest identity and bootstrap support with a CVA4 strain circulating in Shanghai (1047/CVA4/SH/CHN/2010) rather than with CVA6 strains (Fig. 2). In the P3 region, there was no reliable phylogenetic relationship between the recombinant CVA6 and other serotypes of enteroviruses analyzed.

The recombination event was confirmed by the detection of putative breakpoints within genomes using GARD, and further confirmed by statistical analysis of the KH test. The analysis found 5 putative breakpoints, but only the topology flanking position at 4041 bp (*p* = 0.01), which is located within the 2B gene, was significantly discordant via the KH test, supporting that position as a recombination breakpoint (Supplemental Table 3). Best GARD trees based on segment span 3535 to 4041 bp and segment span 4042 to 4470 bp showed marked topologic incongruence. Thus, the result of the GARD analysis was consistent with the Simplot and bootscan analysis results.

### Prevalence of the novel recombinant CVA6 virus among the HFMD cases

To investigate the prevalence of the novel recombinant CVA6 during our observation period and its contribution to the outbreak of CVA6 infection, all of the 292 CVA6 strains were tested by a duplex RT-PCR with two pairs of primers which targeted the non-recombinant and the recombinant CVA6, respectively. This showed that the recombinant CVA6 strain was first detected in November 2012. Among the 292 CVA6 strains, 21.9% (64/292) were typed as the recombinant CVA6 which were identified from 64 throat swabs and 10 vesicle swabs, and 78.1% (228/292) were non-recombinant CVA6 which were identified from 228 throat swabs, 16 vesicle swabs and 1 feces. There was no case that was positive in both PCRs. Since CVA4 could not be detected by CVA4 type-specific RT-PCR (Supplemental Table 4) in the 64 recombinant CVA6-positive specimens, CVA4-like sequences were not likely to have arisen from dual infection with CVA4 and CVA6. Instead, recombination between the two serotypes may explain the findings.

Comparing the two waves of HFMD outbreaks since the novel CVA6 emerged, an increased proportion of the recombinant type in the whole population of CVA6 was observed (9% from November 2012 to January 2013 versus 30.9% from March to September 2013). The proportion of the recombinant type reached its peak (58.8%) in July 2013. This result suggested a partial contribution of the novel CVA6 to the outbreak of CVA6 associated HFMD in Shanghai during 2012 and 2013.

### Phylogenetic analyses of the CVA6 viruses based on VP1, 2C and 3D sequences

**Figure 3:**
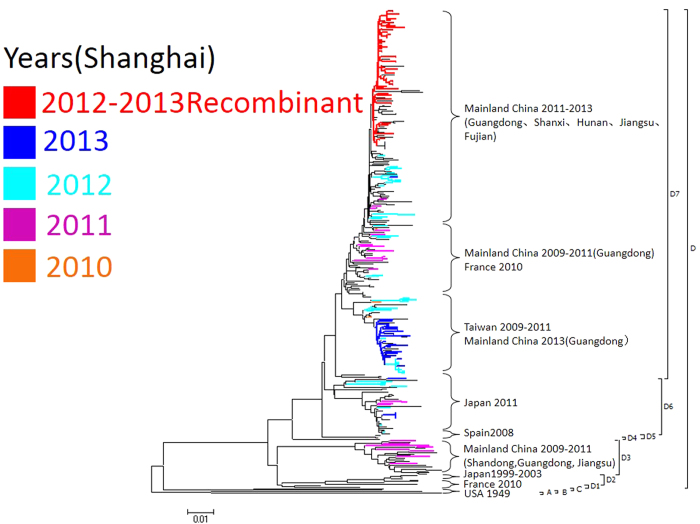
Neighbor-joining trees constructed on the basis of VP1 region of the CVA6 strains by using MEGA 6.05. CVA6 strains isolated from Shanghai are marked with distinct colors according to years of isolation. The clade involving all recombinant CVA6 strains was marked with red color.

The complete VP1 gene was successfully amplified and sequenced for 61 recombinant and 101 non-recombinant CVA6 strains. These 162 CVA6 strains from our study and other CVA6 strains worldwide available from GenBank were subjected to phylogenetic analysis based on full length sequence of the VP1 gene. These strains could be divided into 4 genotypic subgroups, genotypes A, B, C and D, according to the criteria established for genotype EV71 by calculating genetic distance^9^. The CVA6 strains during the CVA6 outbreak period in our study clustered with the strains from other provinces of mainland China, Japan, Taiwan, and France, and made up two predominant clusters, D6 and D7. All recombinant CVA6 isolates together with some of the Guangdong strains isolated in 2012–2013, Hunan in 2012, Jiangsu in 2013, Fujian in 2012 and Shanxi in 2013 clustered in a separated sub-cluster within D7 (Fig. 3; Supplemental Figure 2). This suggested that the recently emerged lineage of CVA6 might circulate in different areas of China, although there is a lack of non-structured protein sequences from these strains.

**Figure 4:**
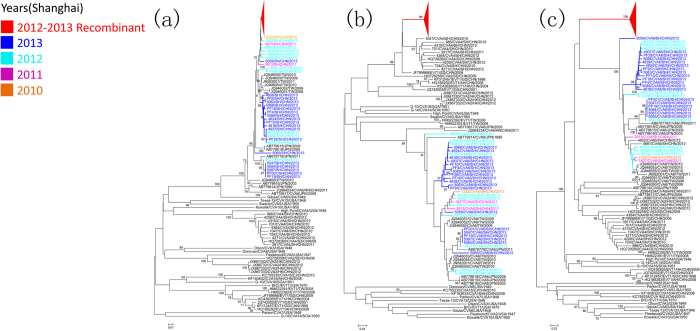
Neighbor-joining trees constructed on the basis of VP1(**a**), 2C(**b**) and 3D(**c**) regions of the recombinant CVA6, non-recombinant CVA6, prototype and recent emerging EV-A strains. by using MEGA 6.05, respectively. CVA6 strains isolated from Shanghai are marked with distinct colors according to years of isolation. Recombinant CVA6 strains are shown by a red triangle. Bootstrap values are indicated for the main branches, in which values lower than 70 are not shown.

The 61 recombinant CVA6 and 33 randomly selected non-recombinant CVA6 strains were amplified and sequenced for full length sequencing of the VP1 gene, and partial 2C and 3D genes. Phylogenetic analysis based on the VP1 gene showed that recombinant CVA6 strains clustered in one clade, which was closely related to the other non-recombinant CVA6 strains (Fig. 4a); however, phylogenetic analysis based on the 2C region showed that recombinant CVA6 strains clustered in one clade, which was closely related to the clade composed of recently emerging CVA4 strains rather than non-recombinant CVA6 strains (Fig. 4b). Based on the 3D region, recombinant CVA6 strains made up a separate branch and showed no close relationship with any specific serotype (Fig. 4c). Thus, phylogenetic analyses supported the findings of the recombinant analysis.

### Clinical features of the HFMD cases infected with this novel recombinant CVA6 virus

Of the 292 patients infected with CVA6, 283 met our criteria and were enrolled in clinical analyses. The demographic and clinical characteristics of the non-recombinant CVA6, and recombinant CVA6 groups are shown in Table 2. We analyzed the relationships of demographic and clinical characteristics between the non-recombinant CVA6 group (n = 223) and the recombinant CVA6 group (n = 60). We found that skin lesions on the upper limbs, lower limbs and anterior trunk were more frequent in the recombinant CVA6 group (*p* = 0.008, *p* = 0.019, *p* = 0.004, respectively) using the chi-square test. The percentages of the generalized form with skin lesions in five or more anatomic sites involved in the non-recombinant CVA6 and recombinant CVA6 groups were 35.4% and 51.7% respectively (*p* = 0.022) using the chi-square test, which indicated that the distribution of skin lesions was more widespread in recombinant CVA6-infected cases. No significant difference was detected in demographic parameters or other clinical or laboratory data between the 2 groups.

## Discussion

Numerous epidemics of HFMD have occurred in the Asia-Pacific region in the past decade, with EV71 the most common cause^7^. Because surveillance of HFMD in mainland China has focused primarily on EV71 and CVA16, information about the pathogenic role of other EVs, and their geographic distribution and epidemiological traits are still limited^6^. Since late 2012, CVA6 has been reported as the predominant pathogen responsible for HFMD in several provinces of China^21^,^23^,^25^,^26^,^27^. Although epidemiologic analyses were performed in those studies, the relationship between molecular characterization and clinical features remain to be demonstrated. In this study, the CVA6 serotype, previously a minor cause of HFMD, was found to be the predominant strain responsible for HFMD in Shanghai since late 2012. Because limited data were available about this emerging virus, we investigated the molecular epidemiology and clinical features of the CVA6 serotype to evaluate the potential risk to public health and to guide in the clinical diagnosis and treatment.

Analyses of complete genomes of EV-A or B species suggested that recombination usually occurred within noncapid regions of the genome^32^,^35^. Multiple EV-A viruses of identical or different serotypes could co-infect an individual and co-circulate in the same geographical area, which gave viruses the opportunity to undergo recombination^22^,^36^,^37^. The co-circulation of CVA4 and CVA6 strains in the same geographic region is a relatively common phenomenon in China^22^,^37^, and would favor the occurrence of recombination between the two serotypes. According to the molecular analyses, the novel CVA6 shared greater similarity with other EV-A strains than with the previously reported CVA6 strains in the partial noncapid 2C region which were most closely related to the CVA4 strains in Shanghai. We believe that recombination probably occurred within the noncapid 2C region between the novel CVA6 virus and a recently emerging CVA4 strain. However, due to no closely related sequences found in GenBank, it was speculated that the P3 region of the recombinant CVA6 may derive from an unknown enterovirus waiting to be discovered.

To clarify the genetic relationship of CVA6 outbreak strains between those in Shanghai and other areas, phylogenetic analysis based on the VP1 gene was performed. Results showed that all CVA6 strains collected during the CVA6 outbreak period in Shanghai together with those from Guangdong, Shanxi, Jiangsu, Fujian and Hunan formed two closely related clusters namely D6 and D7, suggesting that the two subgenotypes had become the major circulating strains in the recent HFMD epidemic in China. All recombinant CVA6 strains in our study clustered together and constitute a monophyletic lineage with some of the Guangdong, Shanxi, Jiangsu, Fujian and Hunan strains within subgenotype D7. This indicates that the recombination event occurred recently and the new recombinant CVA6 isolates together with the strains from other provinces may be descendants of an unknown common recombinant ancestor. Unfortunately, the sequences of other parts of the genomes have not been determined in the CVA6 strains from other provinces. Due to the highly similar VP1 sequences found in the recombinant CVA6 virus in Shanghai and the CVA6 strains from other provinces since the CVA6 outbreak in mainland China, large scale epidemiologic studies throughout the country are warranted to illustrate the role of the novel recombinant CVA6 virus in this HFMD outbreak. Moreover, we must be alert to the possibility that this recombinant CVA6 virus will cause a new HFMD outbreak in the next few years.

The clinical characteristics including the rash distribution among patients infected with distinct serotypes including EV71, CVA6, CVA16 and CVA10 had been compared in previous reports^15^,^25^,^27^,^28^,^37^, so we focus on comparing the clinical characteristics between patients infected with the recombinant and non-recombinant CVA6. Because infection with the recombinant CVA6 virus could result in more generalized skin lesions than infection with non-recombinant CVA6, and the upper limbs, lower limbs and anterior trunk parts were more readily involved in recombinant CVA6-infected cases, dermatologists should pay more attention to this situation in clinical practice.

Our study provides strong evidence that the previously infrequently detected CVA6 virus has become the predominant HFMD pathogen in Shanghai, China, and a novel recombinant CVA6 lineage with special clinical features emerged and partially contributed to the CVA6 outbreak, thus highlighting the necessity of comprehensive surveillance of its circulation in HFMD epidemics in China as well as throughout the rest of the world.

## Additional Information

**How to cite this article**: Feng, X. *et al.* A novel recombinant lineage's contribution to the outbreak of coxsackievirus A6-associated hand, foot and mouth disease in Shanghai, China, 2012-2013. *Sci. Rep.* **5**, 11700; doi: 10.1038/srep11700 (2015).

## Supplementary Material

Supplementary Information

## Figures and Tables

**Table 1:** Pair-wise nucleotide sequence identities between recombinant CVA6 strain and other closely related EV-A strains.

Genomic regions showing discrepant results regarding sequence similarity between recombinant CVA6 (R-CVA6, the representative strain: PF3/CVA6/SH/CHN/2013) and non-recombinant CVA6 (NR-CVA6, the representative strain: 5069/CVA6/SH/CHN/2013) are marked with a gray background. Recombinant EV71 (R-EV71 :JF799986/EV71/GD/CHN/2009), recently emerging CVA4 (E-CVA4:1047/CVA4/SH/CHN/2010), CVA4 prototype strain (P-CVA4:High Point), CVA14 prototype strain (P-CVA14:G-14), CVA16 prototype strain (P-CVA16:G-10) and CVA6 prototype strain (P-CVA6:Gdula) were involved in this analysis.

**Table 2:** Demographic and clinical characteristics of HFMD cases caused by recombinant and non-recombinant CVA6 infections.

*P* < 0.05 is shown in boldface; R-CVA6 represents recombinant CVA6; NR-CVA6 represents non-recombinant CVA6.
