# Supplementary material for: A novel recombinant lineage’s contribution to the outbreak of coxsackievirus A6-associated hand, foot and mouth disease in Shanghai, China, 2012-2013
Source: Sci Rep. 2015 Jun 30;5:11700. doi: 10.1038/srep11700 (PMC4485158; doi:10.1038/srep11700)
Supplement: Supplementary Information [file srep11700-s1.pdf]

Supplementary information

A novel recombinant lineage's contribution to the outbreak of coxsackievirus A6-associated hand, foot and mouth disease in Shanghai, China, 2012-2013

Xiaobo Feng[1]†, Wencai Guan[2]†, Yifeng Guo[1]†, Huiju Yu[3]†, Xiaoling Zhang[2], Ruhong Cheng[1], Zhen Wang[1], Zhen Zhang[1], Jia Zhang[1], Huaguo Li[1], Yin Zhuang[1], Hui Zhang[1], Zhiyong Lu[1], Ming Li[1], Hong Yu[1], Yixiao Bao[3], Yunwen Hu[2], Zhirong Yao[1]

[1]Department of Dermatology, Xinhua Hospital Affiliated to Shanghai Jiaotong University School of Medicine, Shanghai, China

[2]Pathogen Diagnosis and Biosafety Department, Shanghai Public Health Clinical Center, Key Lab of Medical Molecular Virology, Fudan University, Shanghai, China

[3]Department of Pediatrics, Xinhua Hospital Affiliated to Shanghai Jiaotong University School of Medicine, Shanghai, China

Table 1 Primers used in this study.

Oligonucleotide	Sequence (5' to 3')	Position	Purpose
1CA6F	CGGTTAAAACAGCCTGTGGG	1-17	CVA6 genome sequencing
			CVA4 genome sequencing
1268CA6R	CCCGAGCGGTACAAGTAGTG	1249-1268	CVA6 genome sequencing
1113CA6F	TACTCGCCCTGACGTGTC	1113-1130	CVA6 genome sequencing
2059CA6R	GGGAACCAGACCATTGAGTGT	2039-2059	CVA6 genome sequencing
1762CA6F	CTTACAACTGATGACGGGAC	1762-1781	CVA6 genome sequencing
2717CA6R	TTCACCTCCACAACYCCTACYAGC	2694-2717	CVA6 genome sequencing
2333CA6F	TAGACACCCCCACTGAGGCT	2333-2352	CVA6 genome sequencing
			CVA6 VP1 sequencing
3530CA6R	GGCAGTAATACACTCCTGTTTGAC	3530-3553	CVA6 genome sequencing
			CVA6 VP1 sequencing
3265CA6F	CGCCAAACAATAACTAACACTGC	3265-3287	CVA6 genome sequencing
3714CA6F	GTGATTGGTATCGTGTCCACTG	3714-3735	CVA6 2C sequencing
4579CA6R	GCGGAAGTGAGTACACACTAGAGTG	4555-4579	CVA6 genome sequencing
			CVA6 2C sequencing
			CVA4 genome sequencing
4441CA6F	CAGTTCAAGAGCAAACACCGTAT	4441-4463	CVA6 genome sequencing
			CVA4 genome sequencing
5760CA6R	AGGTACAAACATTGAGGGCATG	5739-5760	CVA6 genome sequencing
5642CA6F	CCCTTGACACTAATGAGAAATTCAG	5642-5666	CVA6 genome sequencing
6921CA6R	AGCAACCATGTTGAGTTCATCC	6921-6942	CVA6 genome sequencing
			CVA6 3D sequencing
6493CA6F	GAAGCCAGCAGTTTGAATGACTC	6493-6515	CVA6 genome sequencing
			CVA4 genome sequencing
7400CA6R	GTATAACAAATTTACCCCCACCAGT	7400-7424	CVA6 genome sequencing
			CVA4 genome sequencing
3DCA6F	CGGACCAACTCGCACCAA	6018-6035	CVA6 3D sequencing
3DCA6R	ACCATAAGCAACCATGTTGAGTTC	6925-6948	CVA6 3D sequencing
VP1CA6R	GTAAAACCACTGATAAGCTGTTGC	3019-3042	CVA6 VP1 sequencing
RCA6F	GGCTATAAGCAGCAAGTGGTTAC	4600-4622	R-CVA6 identification
RCA6R	GCGGATGGCATCAGAATCG	4803-4821	R-CVA6 identification
NRCA6F	GGCGTGGATCAAGGCTAAGAC	4047-4067	NR-CVA6 identification
NRCA6R	TCTGGATCTGGCGGAAGTGA	4570-4589	NR-CVA6 identification
ACA4R	CAACTCCAACATCATTAAGGACATC	1204-1228	CVA4 genome sequencing
BCA4F	CGAGTAGATAAACCCACCAGACC	1099-1121	CVA4 genome sequencing
BCA4R	TGTGAAATTCTTTTGCCCTGC	2371-2391	CVA4 genome sequencing
CCA4F	CGTGGTGCCCTGGATAAGTAAC	2217-2238	CVA4 genome sequencing
CCA4R	TGTTATGTGTGGCTAGATGGCG	3431-3452	CVA4 genome sequencing

Oligonucleotide	Sequence (5' to 3')	Position	Purpose
1RCA4F	GATAGACGCAAAATCACTGAAACTG	3318-3344	CVA4 genome sequencing
2FCA4R	ATCACCCACTGGCACAAACAT	5765-5785	CVA4 genome sequencing
3CA4F	GACTCTTGACACCAATGAAAAGTTC	5656-5680	CVA4 genome sequencing
3CA4R	GGCAACCATGTTGAGTTCATCT	6937-6958	CVA4 genome sequencing
A2CA4R	ATTGTCACCATAAGCAGCCA	587-606	CVA4 genome sequencing

R-CVA6 represents recombinant CVA6; NR-CVA6 represents non-recombinant CVA6

Table 2 The information of the 12 reference sequences used in this study

Strain name	Serotype	The place of isolation	The year of isolation	Genbank Accession Number
Fleetwood/USA/1947	CVA2	USA	1947	AY421760
Olson/USA/1948	CVA3	USA	1948	AY421761
High Point/USA/1948	CVA4	USA	1948	AY421762
Swartz/USA/1950	CVA5	USA	1950	AY421763
Gdula/USA/1949	CVA6	USA	1949	AY421764
Parker/USA/1949	CVA7	USA	1949	AY421765
Donovan/USA/1949	CVA8	USA	1949	AY421766
Kowalik/USA/1950	CVA10	USA	1950	AY421767
Texas-12/USA/1948	CVA12	USA	1948	AY421768
G-14/SOA/1950	CVA14	Republic of South Africa	1950	AY421769
G10/SOA/1951	CVA16	Republic of South Africa	1951	U05876
BrCr/USA/1970	EV71	USA	1970	U22521

Table 3 The potential breakpoints deduced by GARD and KH testing report

No. of breakpoints	Breakpoint position (regions of genome)	LHS p-value	RHS p-value	Signficance
1	677	0.00100	0.10400	N.S
2	3534	0.34200	1.00000	N.S
3	4041	0.00100	0.00100	***
4	4470	0.19600	0.49500	N.S
5	4920	0.00100	0.23500	N.S

** for p-value of 0.05; *** for p-value of 0.01

GARD-identified breakpoint via KH test was marked by a gray background.

Table 4 The method for the CVA4 type-specific RT-PCR analysis

Primer	Sequence (5' to 3')	Position	Purpose
CA4F	TATCATAGCAATGGGAGCAGG	2355-2375	CVA4-specific RT-PCR
CA4R	ACTARRCCAGTGTTCAAACTATG	2554-2576	CVA4-specific RT-PCR

The one-step RT-PCR was performed in a final volume of 25 μL containing 2.5 μL genomic RNA, 12.5 μL 2×one step RT-PCR buffer, 0.5 μL TaKaRa Ex Taq HS, 0.5 μL PrimeScript RT Enzyme Mix, 0.5 μL of each primer (10μM), and 8 μL RNase Free dH2O. RT-PCR was performed in an Eppendorf Mastercycler at 42°C for 10min, 95°C for 1 min, followed by 40 cycles of 95°C for 20 s, 56°C for 20 s, and 72°C for 20 s with a final extension step at 72°C for 5 min.

Figure 1 A screen snapshot showing the alignment of recombinant CVA6-specific primer pair (a) and non-recombinant CVA6-specific primer pair (b) with targeted regions, respectively

Figure 2 Neighbor-joining trees constructed on the basis of VP1 region of the CVA6 strains by using MEGA 6.05. CVA6 strains isolated from Shanghai are marked with distinct colors according to years of isolation. The clade involving all recombinant CVA6 strains was marked with red color.

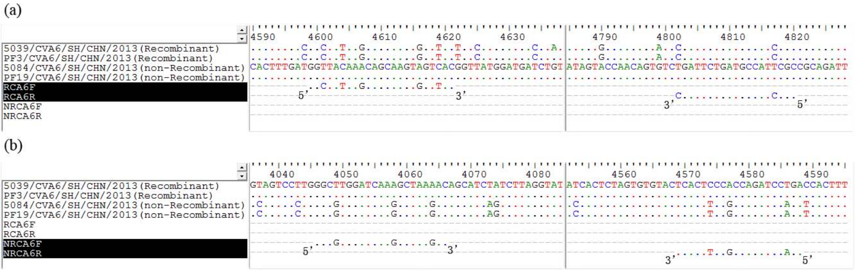
(a)
4590
4600
4610
4620
4630
4790
4800
4810
4820
5039/CVA6/SH/CHN/2013(Recombinant)
PF3/CVA6/SH/CHN/2013(Recombinant)
5084/CVA6/SH/CHN/2013(non-Recombinant)
PF19/CVA6/SH/CHN/2013(non-Recombinant)
RCA6F
RCA6R
NRCA6F
NRCA6R
CACTTTGATGGTTACAAACAGCAAGTAGTCACGGTTATGGATGATCTGT
ATAGTACCAACAGTGTCTGATTCTGATGCCATTCGCCGCAGATT
5'
3'
3'
5'
(b)
4040
4050
4060
4070
4080
4560
4570
4580
4590
5039/CVA6/SH/CHN/2013(Recombinant)
PF3/CVA6/SH/CHN/2013(Recombinant)
5084/CVA6/SH/CHN/2013(non-Recombinant)
PF19/CVA6/SH/CHN/2013(non-Recombinant)
RCA6F
RCA6R
NRCA6F
NRCA6R
GTAGTCCTTGGGCTTGGATCAAAGCTAAAACAGCATCTATCTTAGGTAT
ATCACTCTAGTGTGTACTCACTCCCACCAGATCCTGACCACTTT
5'
3'
3'
5'

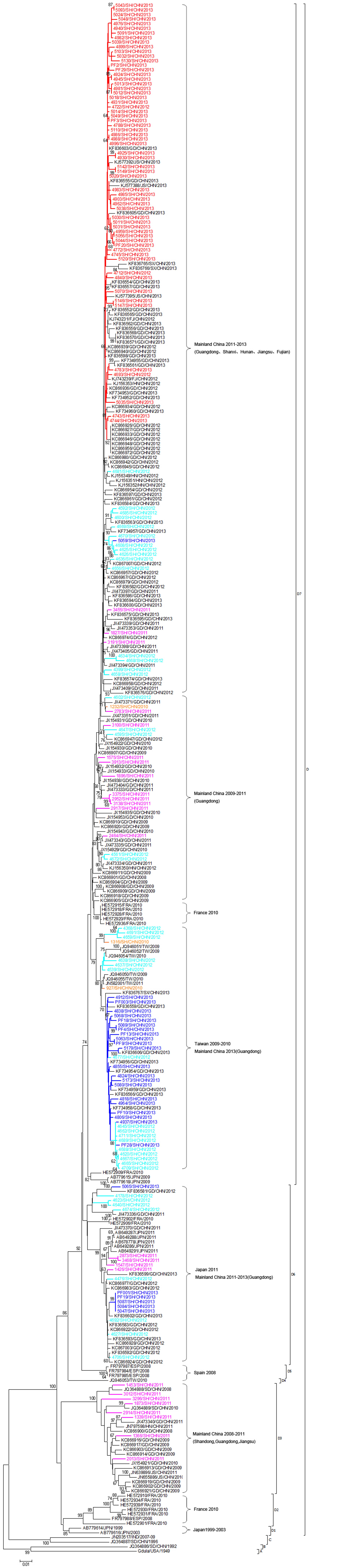
Mainland China 2011-2013
(Guangdong、Shanxi、Hunan、Jiangsu、Fujian)
Mainland China 2009-2011
(Guangdong)
France 2010
Taiwan 2009-2010
Mainland China 2013(Guangdong)
Japan 2011
Mainland China 2011-2013(Guangdong)
Spain 2008
Mainland China 2008-2011
(Shandong, Guangdong, Jiangsu)
France 2010
Japan1999-2003
D7
D6
D5
D4
D3
D2
D1
D
C
B
A
0.01
